# Thiopurine Drugs Repositioned as Tyrosinase Inhibitors

**DOI:** 10.3390/ijms19010077

**Published:** 2017-12-28

**Authors:** Joonhyeok Choi, You-Mie Lee, Jun-Goo Jee

**Affiliations:** Research Institute of Pharmaceutical Sciences, College of Pharmacy, Kyungpook National University, 80 Daehak-ro, Buk-gu, Daegu 41566, Korea; crowz124@naver.com (J.C.); lym@knu.ac.kr (Y.-M.L.)

**Keywords:** cheminformatics, docking simulation, drug repositioning, thiopurine, tyrosinase

## Abstract

Drug repositioning is the application of the existing drugs to new uses and has the potential to reduce the time and cost required for the typical drug discovery process. In this study, we repositioned thiopurine drugs used for the treatment of acute leukaemia as new tyrosinase inhibitors. Tyrosinase catalyses two successive oxidations in melanin biosynthesis: the conversions of tyrosine to dihydroxyphenylalanine (DOPA) and DOPA to dopaquinone. Continuous efforts are underway to discover small molecule inhibitors of tyrosinase for therapeutic and cosmetic purposes. Structure-based virtual screening predicted inhibitor candidates from the US Food and Drug Administration (FDA)-approved drugs. Enzyme assays confirmed the thiopurine leukaemia drug, thioguanine, as a tyrosinase inhibitor with the inhibitory constant of 52 μM. Two other thiopurine drugs, mercaptopurine and azathioprine, were also evaluated for their tyrosinase inhibition; mercaptopurine caused stronger inhibition than thioguanine did, whereas azathioprine was a poor inhibitor. The inhibitory constant of mercaptopurine (16 μM) was comparable to that of the well-known inhibitor kojic acid (13 μM). The cell-based assay using B16F10 melanoma cells confirmed that the compounds inhibit mammalian tyrosinase. Particularly, 50 μM thioguanine reduced the melanin content by 57%, without apparent cytotoxicity. Cheminformatics showed that the thiopurine drugs shared little chemical similarity with the known tyrosinase inhibitors.

## 1. Introduction

The colour of the human skin is predominantly determined by the amount of melanin that is produced in the skin melanocytes. Dark-coloured eyes and hair, as well as browning of food, are also related to elevated melanin content. The enzymatic reaction of tyrosinase is the primary process in melanin production in living organisms. The cognate substrate, tyrosine, is converted into dihydroxyphenylalanine (DOPA) and subsequently into dopaquinone by tyrosinase, and then spontaneously into melanin via eumelanin. Tyrosinase, catechol oxidase, and haemocyanin are type-3 copper proteins, which possess two juxtaposed copper ions in the catalytic centre. Three evolutionarily conserved histidine residues form coordinate bonds with a copper ion. In the hydroxylation of monophenol to diphenol, and its subsequent conversion to quinone by tyrosinase, catechol oxidase only catalyses the second oxidation. In contrast, the primary role of haemocyanin in some invertebrates is the carriage of oxygen. The copper ions of tyrosinase interchange among four oxidation states (oxy-, met-, deoxy-, and deact-) [1]. The catalytic activity is coupled to the cycle, providing the substrates with molecular oxygen.

The uncontrolled activity of tyrosinase causes disorders. For example, albinism is a congenital disorder, in which the body scarcely synthesises melanin, owing to the absence of tyrosinase, whereas overactivation of tyrosinase increases melanin synthesis, which causes skin problems. Abnormalities in tyrosinase activity have also been associated with other diseases, such as cancer and Parkinson’s disease [2,3,4,5]. Efforts to discover and develop small molecules that selectively modulate the function of tyrosinase for the treatment of skin conditions have continued, including the development of skin-lightening cosmetics and products for agricultural purposes [6,7,8,9,10,11,12,13]. Tyrosinase inhibitors can be largely divided into two classes: polyphenolic compounds and thiourea derivatives [14]. The polyphenolic compounds are natural-based, such as glycosylated hydroquinones from plants and arbutin, a skin-lightening agent. Phenylthiourea and its analogues constitute the other class.

The crystal structures of the complexes between two substrates, tyrosine and DOPA, and bacterial tyrosinase show snapshot transition structures under conditions where zinc atoms replace the copper atoms [15]. The structures revealed a subtle variation in the orientations of tyrosine and DOPA, which facilitates the understanding of the differences in tyrosinase and catechol oxidase. In contrast, the relevant complex structures with inhibitory effects are limited in number, which makes it difficult to obtain a detailed explanation of tyrosinase inhibition. Three polyphenolic compound complexes, one between tropolone and mushroom tyrosinase [16], and the other two between kojic acid and hydroquinone and bacterial tyrosinase [17], are available. In addition, the structures of complex formed between catechol oxidase and phenylthiourea has provided insight into another type of inhibition [18]. These structures underscore that the binding of inhibitors to tyrosinase does not result in the oxy-state, but rather the met-state, where the oxygen atoms of tropolone, kojic acid, and hydroquinone, and the sulphur of phenylthiourea replace the bridging oxygen atoms that exist in the apo-state. Nonetheless, the lower sequence identity between mushroom and mammalian tyrosinases (<25%), and the lack of the study with purified animal tyrosinase at molecular level makes the generalization obscure. For instance, the recent study reported the large differences in the quantified inhibition dependent on the originating species of tyrosinase [14,19]. This is difficult to explain with the currently available information. The different cellular locations and states in mushroom and mammalian tyrosinases may hinder the clear explanation even more [6,9].

Drug repositioning is defined as the application of the existing drugs to new uses. Repositioning can reduce the time and cost that is required for the typical drug discovery process. Accumulated pharmaceutical and toxicological data have uncovered the polypharmacological networks of numerous drugs. Nevertheless, effective strategies for drug repositioning, particularly those that are amenable to academic laboratories, are less well established. Computational methods can significantly facilitate drug repositioning [20,21,22,23,24,25,26,27]. We previously reported cheminformatics-based drug repositioning, which resulted in the identification of ethionamide and its analogues [28], and thiourea-containing drugs [29] as novel inhibitors of tyrosinase. In this study, we identified additional agents for repositioning as new tyrosinase inhibitors using a structure-based virtual screening (SBVS). Imperfections are present in the SBVS, as exemplified by the improper handling of protein flexibility and solvent molecules. Nevertheless, it has been successfully used in the drug discovery process as a complementary strategy to high-throughput screening [30]. SBVS can enrich the true-positives and decrease the number of test molecules to a manageable range using careful assays. We reported an optimised pairing of structures and software that led to the discovery of new classes of potent tyrosinase inhibitors [31]. In this study, we have employed the pairing for SBVS-based drug repositioning.

## 2. Results

### 2.1. Structure-Based Docking Simulation Revealed Thioguanine as a Tyrosinase Inhibitor

**Figure 1:**
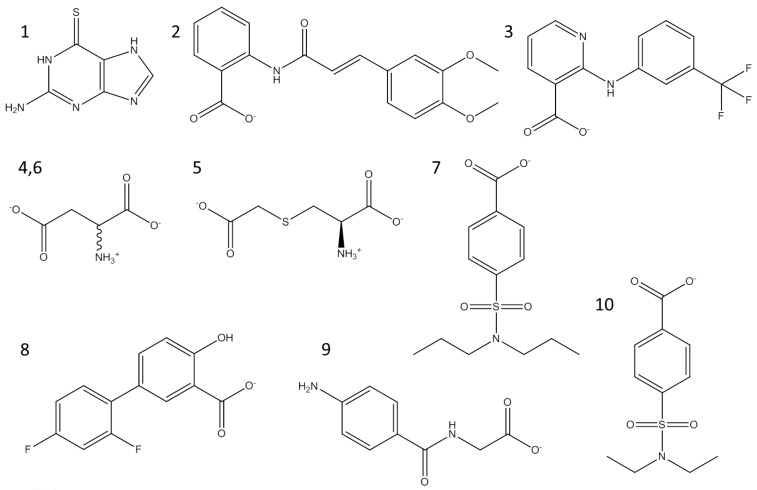
Top 10 candidate molecules predicted as potential tyrosinase inhibitors from the US Food and Drug Administration (FDA) subset of ZINC database. Molecules **1**–**10**, which showed the lowest DOCK 3.6 scoring function were thioguanine (ZINC18085533, scoring function: −69.3 kcal/mol), tranilast (ZINC797, −64.7), nifumic acid (ZINC125031, −62.3), d-aspartic acid (ZINC895218, −61.9), S-carboxymethyl-l-cysteine (ZINC1529732, −60.2), l-aspartic acid (ZINC895032, −60.0), probenecid (ZINC1982, −59.2), diflunisal (ZINC20243, −59.1), aminohippuric acid (ZINC119344, −58.9), and etebenecid (ZINC1380, −58.4). d,l-aspartic acids (**4**,**6**) were drawn in a chemical structure.

**Figure 2:**
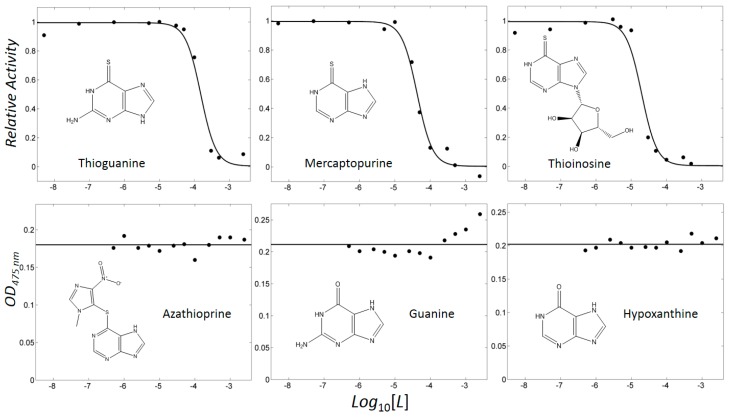
Profiles showing concentration-dependent inhibition. Activities were scaled to a relative value in the range of 0–1 for thioguanine, mercaptopurine, and thioinosine. Optical densities at 475 nm (OD_475nm_) were shown for azathioprine, guanine, and hypoxanthine.

In our previous study, the optimised pairing of an algorithm and three-dimensional (3D) structures considerably improved the prioritisation of true-positives in the high-throughput virtual screening of inhibitors of mushroom tyrosinase [31]. The reproduced poses of phenylthiourea and tropolone were compared with those that were found in the crystal structures, leading to the selection of DOCK 3.6 [32] as the docking algorithm among four software. Dockings with the molecular dynamics simulation-derived ensemble using a mixture of known ligands and their physicochemically matched, but topologically different, decoys led to the selection of the best structure based on the metrics from the receiver operating characteristic (ROC) curve. We then investigated whether the pair could facilitate drug repositioning. We used an identical pair of algorithms and structures to screen a subset of the ZINC12 database [33,34,35], the US Food and Drug Administration (FDA)-approved drugs that comprises 3182 small molecules. The top 10 compounds that showed the lowest energy were thioguanine (ZINC18085533), tranilast (ZINC797), nifumic acid (ZINC125031), d-aspartic acid (ZINC895218), s-carboxymethyl-l-cysteine (ZINC1529732), l-aspartic acid (ZINC895032), probenecid (ZINC1982), diflunisal (ZINC20243), aminohippuric acid (ZINC119344), and etebenecid (ZINC1380), in ascending order of their DOCK 3.6 energies (Figure 1). Interestingly, an extensive literature survey found that the inhibition of tyrosinase by diflunisal and aminohippuric acid through direct binding has been reported [36,37]. However, there has been no report on whether the other eight molecules inhibit tyrosinase. Therefore, we tested whether thioguanine, tranilast, nifumic acid, d,l-aspartic acid, s-carboxymethyl-l-cysteine, and probenecid could inhibit mushroom tyrosinase, but only thioguanine exerted inhibitory action (Figure 2). The quantified inhibition expressed as the inhibitory constant (*K_i_*) was 52 μM (Table 1). It is noteworthy that the enzyme assay experiments included 0.01% Triton X-100 to exclude false-positives that were caused by colloidal aggregation [38]. When considering the chemical similarity between probenecid and etebenecid, it would be reasonable to assume that etebenecid also has no inhibitory effect.

### 2.2. Mercaptopurine and Thioinosine Inhibited Tyrosine Activity

**Figure 3:**
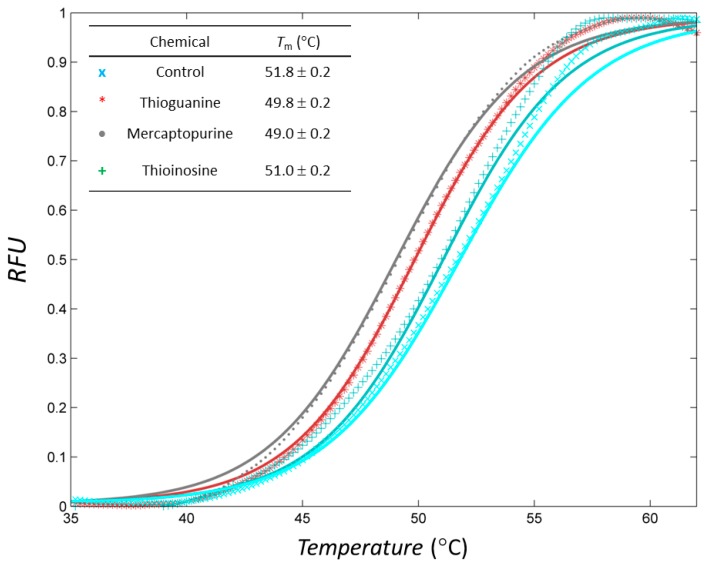
Profiles of differential scanning fluorimetry (DSF) analyses of inhibitors. Markers and lines indicate the raw and fitted data, respectively, in each case. Normalised profiles with relative fluorescence unit (RFU) were constructed in the range of 35–65 °C.

Thioguanine, also called tioguanine or 6-thioguanine, is a drug for the treatment of leukaemia. It is one of the essential medicines that the World Health Organization specifies are required for a basic health system. In addition to thioguanine, mercaptopurine (ZINC4658290) and azathioprine (ZINC4258316) comprise the thiopurine family of drugs. However, in contrast to thioguanine and mercaptopurine, immunosuppression is the primary indication of azathioprine. We evaluated whether mercaptopurine and azathioprine inhibit tyrosinase, and found that mercaptopurine, but not azathioprine, exerted an inhibitory activity (*K_i_* = 16 μM, Figure 2). This suggests that the sulphur atom possibly plays a significant role in the interaction with tyrosinase. To confirm this hypothesis, we conducted enzymatic assays with guanine (ZINC895129), hypoxanthine (ZINC36378435), and thioinosine (also known as mercaptopurine-riboside, ZINC4217548). Guanine and hypoxanthine are analogues of thioguanine and mercaptopurine, respectively, in which oxygen atoms replace sulphur atoms. The metabolism of mercaptopurine generates thioinosine in humans through the attachment of sugar at the opposite position to that of sulphur in purine. Of these molecules, only thioinosine inhibited tyrosinase (*K_i_* = 8 μM, Figure 2). These results were consistent with our previous data that emphasised the importance of sulphur in ethionamide and thiourea-containing drugs for the inhibition of tyrosinase [28,29]. The quantified value of the tyrosinase inhibition by mercaptopurine was comparable to that of kojic acid (13 μM, Figure S1), which is a well-known and potent tyrosinase inhibitor, under the same experimental conditions (Table 1). We measured the change in *T*_m_ value using differential scanning fluorimetry (DSF) to confirm direct binding. An increase or decrease in the *T*_m_ value reflects the stabilisation or destabilisation of a protein following direct binding with inhibitors, respectively. DSF showed that there was a shift in the *T*_m_ values of tyrosinase from 51.8 °C to 49.8, 49.0, and 51.0 °C in the complexes with thioguanine, mercaptopurine, and thioinosine, respectively (Figure 3). The degrees of change (Δ*T*_m_) in the three inhibitors were all statistically significant (*p* < 0.01). A decrease in this value is indicative of the destabilisation of the protein following its direct binding with the inhibitors.

### 2.3. Enzyme Inhibitory Kinetics Classified Thiopurine Drugs as Competitive Inhibitors

**Figure 4:**
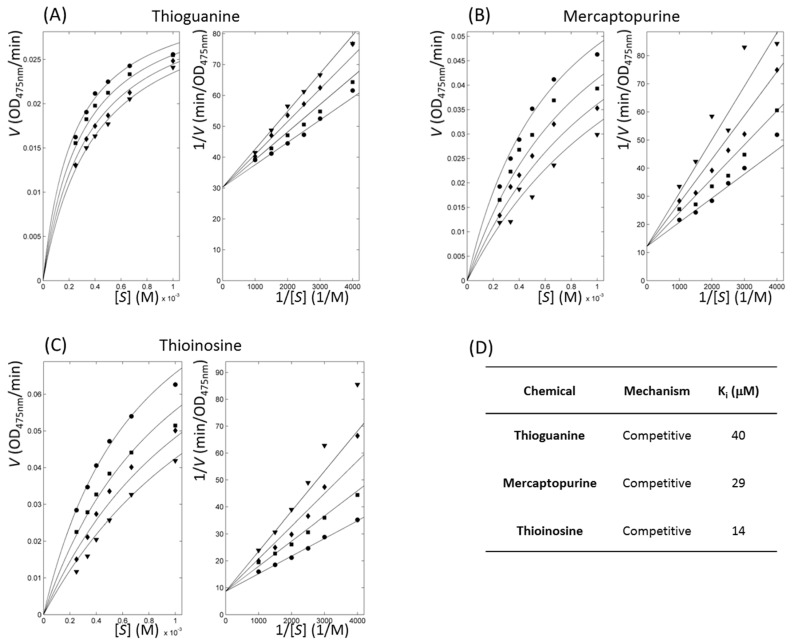
Inhibitory enzyme kinetics with tyrosinase inhibitors. (**A**–**C**) Left and right panels show Michaelis–Menten and Lineweaver–Burk plots, respectively for each labelled compound. Respective symbols, ●, ■, ◆, and ▼ represent inhibitor profiles at concentrations of 90, 120, 150, and 180 μM for thioguanine; 20, 40, 60, and 80 μM for mercaptopurine; and 10, 20, 30, and 40 μM for thioinosine. (**D**) Parameters of enzyme inhibitory kinetics are listed. *K_i_* indicates fitted dissociation constants of the protein-inhibitor complex using nonlinear data fitting, assuming a competitive model.

The kinetics of enzymes with inhibitory action classified thioguanine, mercaptopurine, and thioinosine as competitive inhibitors. We directly fitted all of the substrate and inhibitor concentration-dependent velocities using the modified Michaelis–Menten equation in a nonlinear manner through the minimisation of χ^2^ values in four kinetic models: competitive, uncompetitive, non-competitive, and mixed [40,41] (Figure 4). Among the competitive, uncompetitive, and non-competitive models, the competitive model showed the smallest values for the three compounds. The values of reduced χ^2^, χ^2^ divided by the degree of freedom for thioguanine, mercaptopurine, and thioinosine in the competitive model were 0.08, 0.91, and 0.43, respectively. The *F*-test showed that no statistical significance occurred in the mixed model when compared to the competitive model. The assigned models were sound when considering that the three molecules share functional moieties. The qualitative agreement of the experimental and fitted inhibitory constant, *K_i_*, also supported the soundness of the analyses. The kinetically fitted *K_i_* values for thioguanine, mercaptopurine, and thioinosine were 40, 29, and 14 μM, respectively. It is noteworthy that in this situation, the apparent kinetics was observed. The underlying mechanism would be more complicated, and the limited precision of the current dataset would make it difficult to extract the details [42].

### 2.4. Thiopurine Drugs also Inhibited Mammalian Tyrosinase

**Figure 5:**
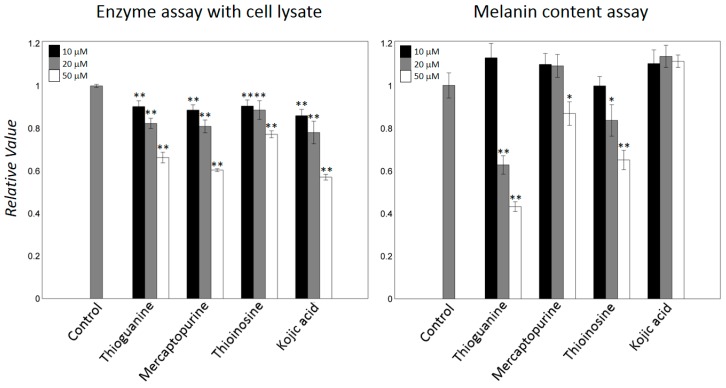
Mammalian tyrosinase inhibition. B16F10 cell lysates were used to evaluate inhibition against different concentrations of mammalian tyrosinase inhibitors. Melanin contents were measured at concentrations that exerted only a small effect on cell growth of B16F10 cells. Data are expressed as relative values to those of untreated control cells. Error bars indicate standard deviations of three repeated experiments per each condition. The * and ** were labelled when the differences are statistically significant at the levels of *p* < 0.05 and *p* < 0.01, respectively, compared with the controls by *t*-test.

To determine whether the thiopurine-drugs inhibit mammalian tyrosinase, we performed an enzymatic assay with melanoma B16F10 cell lysates. The addition of thioguanine, mercaptopurine, and thioinosine to the lysates inhibited melanin syntheses in a concentration-dependent manner. Mercaptopurine exhibited the highest inhibition, showing a comparable decrement to that induced by kojic acid (Figure 5). The cytotoxicity and the cellular activity of thioguanine, mercaptopurine, and thioinosine were assessed using the 3-(4,5-dimethylthiazol-2-yl)-2,5-diphenyltetrazolium bromide (MTT) and melanin content assays, respectively, in B16F10 cells. Previous results showed that thioguanine and mercaptopurine are less cytotoxic up to several tens of μM in human hepatocytes [43]. Although we cannot exclude other potential cellular damages, the addition of up to 50 μM of any of the three inhibitors did not notably decrease the reduction in the MTT reaction. Thioguanine and thioinosine markedly reduced the melanin content at 20 and 50 μM, whereas mercaptopurine was less effective (Figure 5). In particular, the quantification of the decrease that was caused by 50 μM thioguanine showed that it was approximately 57%. Meanwhile, different from the enzyme inhibition with cell lysate, the decrement of melanin contents by kojic acid was marginal under the tested conditions. It is consistent with the data that reported the meaningful decreases by kojic acid at higher concentrations (>100 μM) [44,45,46]. There was no obvious correlation in the results by enzyme- and cell-based assays. Several possibilities may explain the inconsistencies. First, the structural difference between mushroom and mammalian tyrosinases can result in the varied inhibitions. The sequence identity between two tyrosinases is smaller than 25% although the residues comprising the catalytic core are evolutionarily conserved (Figure S2). Second, the existences of the bindings to off-targets can cause the differences in the active concentrations. Third, pharmaceutical features, such as cell penetration and solubility under cellular environments, may differ in the three inhibitors.

### 2.5. Docking Simulation Suggested Intermolecular Interactions Involved Atomic Contributions

**Figure 6:**
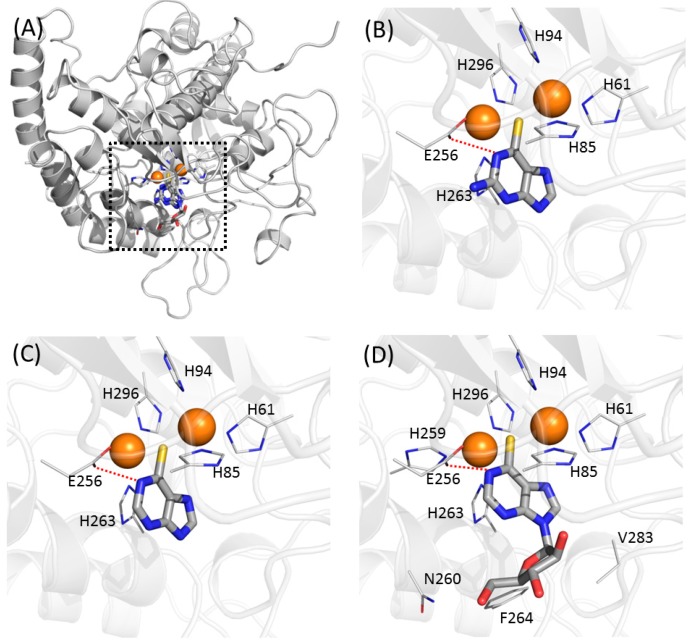
Predicted binding modes of thiopurine inhibitors in this study. (**A**) Overlaid poses against mushroom tyrosinase. Two copper atoms in tyrosinase are indicated as orange spheres. Predicted binding modes in (**B**) thioguanine, (**C**) mercaptopurine, and (**D**) thioinosine are represented. Residues involved in intermolecular contacts from tyrosinase are labelled. Lines indicate intermolecular hydrophilic interaction. Figures were prepared and arranged to have identical direction using Pymol [47].

In the modelled structures, the thiopurine moieties of thioguanine, mercaptopurine, and thioinosine almost completely overlapped (Figure 6). Of the four inhibitors (tropolone, kojic acid, hydroquinone, and phenylthiourea (Figure S4) reported as complex structures with tyrosinase or catechol oxidase, phenylthiourea possessed a similar functional moiety to that of current inhibitors [16,17,18]. The limited sequence identity between mushroom tyrosinase and catechol oxidase (<20%) makes the direct interpretation of the binding mode of phenylthiourea to mushroom tyrosinase equivocal. Nonetheless, it can be assumed that the position of the functional moiety does not change between the two structures. The sulphur atom of the thiopurine moiety lies at an almost identical position to that of phenylthiourea, bridging two copper atoms. The distances between the two copper atoms and the sulphur of phenylthiourea in the crystal structures were 2.26 and 2.33 Å, and those in the docked thioguanine were 2.50 and 2.56 Å, reflecting the similarity of the two bound poses (Figure 6). However, a difference also exists between the structure of the phenylthiourea-catechol oxidase complex and the docked models of thiopurine-tyrosinase. While the nitrogen between the thione and phenyl groups of phenylthiourea is in contact with a copper atom in the crystal structure [18], the very next nitrogen to the thione group of thiopurine forms an intermolecular hydrogen bond with the Oε1/2 of Glu-256 in the models (Figure 6). The discrepancy may be attributed to the imperfections in the docking algorithm, or to the difference in the protein and inhibitor. New 3D structures of tyrosinase in complex with inhibitors would be necessary to provide an unambiguous explanation. In thioinosine, additional contacts with the side-chains of Asn-260, Phe-264, and Val-283 were observed, which was thought to have caused the stronger inhibition. The sequence alignment with mushroom and human tyrosinases shows that the five contacting residues with thiopurine inhibitors (His-61, His-85, His-94, Glu-256, and His-263) have the corresponding amino acids, despite the remote sequence identity (Figure S2). Except for the position of 256, where human tyrosinase has Ser, the others are evolutionarily conserved. It may provide insight for explaining the activities in mammalian cells. However, we cannot currently exclude the inhibition through another mechanism. The docking simulation with the E256S mutant resulted in more diverse poses than those with wild-type, although the poses share the apparent similarities (Figure S3). In particular, the intermolecular hydrophilic contact with the residue of 256 disappear in mercaptopurine, whereas thioguanine and thioinosine possess the contacts within 3.8 Å. Our models would be a suggestive clue for the future biophysical studies at the individual residue level.

### 2.6. Cheminformatics Supported Novel Chemical Structures in Thiopurine Tyrosinase Inhibitors

**Figure 7:**
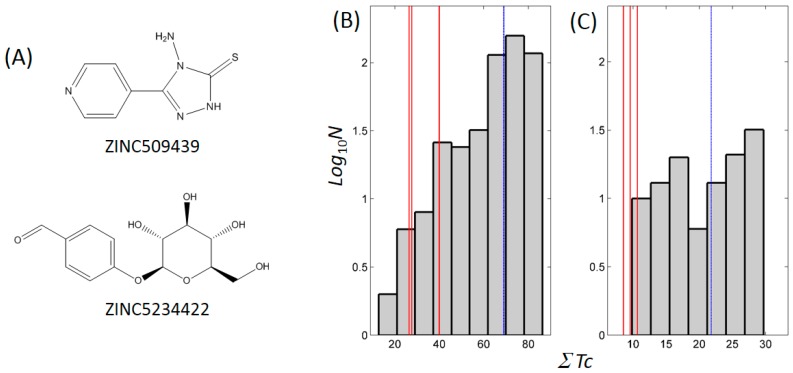
Quantification of chemical similarity to known tyrosinase inhibitors. (**A**) Of the 418 BindingDB-deposited known inhibitors [48], the most similar ones to the inhibitors in this study are presented. ZINC509439 showed the closest chemical structure to thioguanine and mercaptopurine with Tanimoto coefficients (*Tc*) of 0.15 and 0.18, respectively, whereas the closest inhibitor to thioinosine was ZINC509439 with a *Tc* of 0.27. Histograms of distributions of ∑*Tc* values of (**B**) all and (**C**) 115 thione-containing tyrosinase inhibitors are presented. ∑*Tc* is defined as the sum of *Tcs* between a test molecule and a set of inhibitors. *N* in *y*-axes means the frequency. ∑*Tc* values of thioguanine, mercaptopurine, and thioinosine were (**B**) 27.62, 26.45, and 39.95 and (**C**) 9.58, 8.56, and 10.68, respectively. Corresponding positions are shown as red lines. Mean ∑*Tc* values in all and 115 thione-containing tyrosinase inhibitors were 69.00 and 21.81, respectively, and are denoted as dashed blue lines.

We searched the chemically similar known inhibitors. BindingDB (2015m6 version) [48] contains 486 small molecules that inhibit tyrosinase through direct binding from all species. Among the molecules, the most similar inhibitor to thioguanine and mercaptopurine was 4-amino-5-(4-pyridyl)-4H-1,2,4-triazole-3-thiol (ZINC509439), with Tanimoto coefficient (Tc) values of 0.15 and 0.18, respectively (Figure 7A). The reported K_i_ value of the compound was 1 μM. In contrast, 4-(β-d-allopyranosyloxy)benzaldehyde (ZINC5234422) showed the closest similarity to thioinosine, with a *Tc* value of 0.27 and a half-maximal inhibitory concentration (IC_50_) of 94 μM. The smaller *Tc* value indicated that the thiopurine drugs showed limited chemical similarities to the known inhibitors, which was supported by the apparent differences. To assess the limited similarity in detail, we used the similarity ensemble approach (SEA) [49], which involves summing up the Tc values between a test molecule and a set of small molecules to obtain the ∑*Tc*. The histogram of the ∑*Tc* values between a set of inhibitors shows the distribution of chemical similarities. When comparing the ∑*Tc* value of a test molecule and the distribution can demonstrate the chemical novelty of a test molecule. A molecule that has greater chemical similarity to the others would exhibit a higher ∑*Tc* value. The ∑*Tc* values were 27.62, 26.45, and 39.95 for thioguanine, mercaptopurine, and thioinosine, respectively (Figure 7B). The values were ranked as number 480, 482, and 467 of the 486 tyrosinase inhibitors from the highest score molecule, ZINC28645001, which is a 2,4-resorcinol derivative and has a ∑*Tc* value of 93.87 [50]. Owing to the central roles and the frequent use of DNA bases in living organisms and its close similarity to guanine, thioguanine has been reported as a direct binder of various cellular proteins. A literature search of the ChEMBL database [51] revealed that thioguanine (ID: CHEMBL727) inhibited the functions of p21 (RAC1) activated kinase 1 (PAK1) [52], 6-oxopurine phosphoribosyltransferases [53], human xanthine oxidase [54], uridine nucleoside ribohydrolase [55], and monoamine oxidase (MAO)-A,B [56] through direct binding. As purine is the core moiety responsible for the effects of these proteins, both guanine and thioguanine are inhibitory. Indoleamine 2,3-dioxygenase 1 is one of the known targets of mercaptopurine (CHEMBL1425) and is a protein that is inhibited by thioguanine [57]. A previous study reported the interaction between thioinosine (CHEMBL448290) and MAO-A/B [56]. However, to the best of our knowledge, no known target proteins that are related to skin pigmentation have been reported.

## 3. Discussion

Several cases of drug repositioning used SBVS, which is primarily targeted at discovering inhibitors of new chemotypes, which cannot be identified using quantitative structure-activity relationship analyses [20,21,22,23,24,25,26,27]. The limited similarity to the known inhibitors may hinder the repositioning of thiopurine drugs as tyrosinase inhibitors using cheminformatic methods. Even SBVS, which is a conventional approach, was insufficient for the repositioning of thioguanine. The use of the optimised pair of structure and algorithm was indispensable. Specifically, the six coordinates from the two crystal structures of mushroom tyrosinase (2Y9W and 2Y9X) [16] generated different candidates, even with the same protocol and database. In the structures of 2Y9W-A (PDB ID-Chain), 2Y9W-B, 2Y9X-A, 2Y9X-B, 2Y9X-C, and 2Y9X-D, the rankings of docked thioguanine were 11, 13, 24, 21, 25, and 17, respectively. Although we cannot exclude the possibility that the highly ranked candidates are inhibitory as well, the results clearly demonstrate the dependence of the scoring function in SBVS on the template structure and the need to select the best structure and docking algorithm pair. It should be noted, that software programs other than DOCK 3.6, AutoDock, AutoDock Vina, and DOCK 6.7, were unsuccessful in generating the poses of tropolone or phenylthiourea that were observed in X-ray structures in our previous study [31]. The portion of the considered molecules by enzyme assay corresponds to about 0.3% (10/3182) in the current study. The value is much higher when compared to the portion of the tested compounds in the conventional high-throughput SBVS (HTSBVS). The median value of the tested molecules in the 52 cases of HTSBVS was 26 [30]. Here, a case of HTSBVS docked more than several hundreds of thousands of molecules on average, implying that less than 0.03% of total molecules were tested.

The inhibitory effects of the compounds against tyrosinase may not be deemed as strong. However, it is noteworthy that mercaptopurine had a ligand efficiency (LE) value of 0.61 (Table 1). In the drug discovery process, an LE value > 0.4 is a good criterion for determining the feasibility of further developing a hit into a lead compound [39]. The LE values of thioguanine and thioinosine were 0.49 and 0.34, respectively. The stronger inhibition induced by mercaptopurine than by thioguanine is difficult to explain using the current docking models because both molecules have almost identical binding poses. One possible explanation is the steric hindrance that could arise from the amide group in thioguanine. The different electrostatic charges around the metal binding region may be responsible for the difference.

The small molecule inhibitors of metalloenzymes generally possess a metal-binding group that forms the coordinate bonds with catalytic metal cofactors. Our previous experiments classified the metal-binding motifs in tyrosinase inhibitors into four types: carboxylate, thione, triazole, and tetrazole [31]. In addition, some polyphenol compounds can inhibit tyrosinase by mimicking the substrate. In the current study, thioguanine was included in the thione group. The other molecules in the 10 candidate drugs that were identified by SBVS included carboxylates. Nevertheless, the six tested molecules hardly inhibited mushroom tyrosinase, which indicates that the existence of the metal-binding group is a necessary but not sufficient condition. The cheminformatics analysis revealed that 115 molecules of BindingDB-derived 486 inhibitors contain a thione moiety (Figure S4). Only four molecules possess thiones, to which no nitrogen is bound. One molecule (ZINC2573195) can be classified as a polyphenol, and the others (ZINC1680424, ZINC1706841, and ZINC2019878) belong to xanthate [58], which is known as a typical metal chelator. All the other thione-containing inhibitors contain nitrogen that can be used for the intermolecular electrostatic contact. However, the thiopurine inhibitors in the current study showed little similarity to other thione-containing tyrosinase inhibitors. There is no inhibitor that possesses both purine and thione moieties. The ∑*Tc* values against the 115 thione-containing molecules were 9.58, 8.56, and 10.68 for thioguanine, mercaptopurine, and thioinosine, respectively (Figure 7C). No inhibitor showed a smaller ∑*Tc* than that of thioguanine and mercaptopurine, whereas thioinosine was ranked as number 113.

Taken together, we demonstrated an SBVS-based drug repositioning strategy where the sophisticated combination of the docking algorithm and the template structure successfully enriched the inhibitory compounds for tyrosinase from FDA-approved drugs. Accompanying enzyme- and cell-based assays confirmed and quantified the inhibitory activities of thiopurine anticancer drugs. Computational tools characterized the distinctness in the chemical structures of the thiopurine tyrosinase inhibitors. Our approach represents a useful strategy to drug repositioning methods by using computational tools. Moreover, new inhibitors and their proposed inhibitory mechanisms in this study will be valuable information for the development of more potent and selective inhibitors of metalloenzymes.

## 4. Materials and Methods

Structure-based virtual screening—An in-house script, Automated pLatform for Integrative Structure-based DOCKing (ALIS-DOCK), was prepared and used to perform the SBVS with the molecules from a subset of ZINC12 database named “FDA-approved drugs (via DSSTOX)” [35]. For the docking engine, ALIS-DOCK used DOCK 3.6 [32]. ALIS-DOCK used the structure chosen for the structure-based high-throughput virtual screening as a template. The protocol for docking was identical in principle to that of the DOCK Blaster [59]. Molecules in the flexibase format with ligand desolvation scoring terms [32] were directly adapted from the ZINC database [35]. The scoring function of DOCK 3.6 is the sum of the van der Waals and electrostatic energies corrected by the desolvation terms. The charge for copper atoms was reduced from 1.40 to 0.73, based on the quantum chemical calculations [31].

Cheminformatics—The *Tc* was used to quantify the similarity between two small molecules. The Morgan circular fingerprints that were implemented in RDKit (http://www.rdkit.org) digitise the functional moieties in a molecule. *Tc* evaluates the common over the union digitised features between two molecules and is a value between 0 and 1. Two chemicals sharing no and complete overlap show *Tc* values of 0 and 1, respectively. Inhibitors of tyrosinase from all species were extracted from the BindingDB database [48]. The in-house script, Automated LIgand Search for PolyPharmacology (ALIS-PP) was used to automate the cheminformatics procedure.

Enzyme activity and kinetics experiments with inhibitors—All of the reagents used in this study were purchased from ChemBridge (San Diego, CA, USA), Sigma-Aldrich (St. Louis, MO, USA), or Tokyo Chemical Industry (Tokyo, Japan). The reaction solution for the enzyme assay was prepared with 200 nM mushroom tyrosinase and inhibitors in phosphate-buffered saline (PBS), containing 5% dimethyl sulphoxide and 0.01% (*w*/*v*) Triton X-100. After incubating the mixture at 30 °C for 10 min, 500 μM l-tyrosine was added as the substrate. Subsequently, the absorbance change induced by the chromogenic product, dopachrome, was measured at 475 nm. The IC_50_ was evaluated by analysing different concentrations of each inhibitor. The inhibitory constants (*K_i_*) were converted from the IC_50_ values using the *K_m_* obtained from kinetics experiments and substrate concentration based on the Cheng–Prusoff equation [60]. A series of substrates at concentrations of 0.25, 0.33, 0.4, 0.5, 0.67, and 1 mM were used to obtain the respective velocities (*V*) for enzyme inhibitory kinetics. The concentrations of each inhibitor were varied and comprised the range that included the IC_50_ value. Four models (competitive, uncompetitive, non-competitive, and mixed) that were derived from the Michaelis-Menten equation were used [61] to fit the kinetic parameters, maximum velocity (*V_max_*), Michaelis constant (*K_m_*), and the dissociation constants between the substrate-free enzyme and inhibitor (*K_ic_*) and the substrate-bound enzyme and inhibitor (*K_iu_*). The data from all the concentrations of the substrate and inhibitor were simultaneously fitted nonlinearly by minimising the χ^2^ value, defined as the sum of the squared deviations between experimental and fitted values [40] for each model. The appropriate model was selected based on the *F*-test with χ^2^ values from the four models, coupled with the extraction of the related parameters [40,62]. All of the fittings and statistical analyses in this study were computed by using MATLAB^®^ (MathWorks, Natick, MA, USA).

DSF—The stability of mushroom tyrosinase in the presence and absence of inhibitors was assessed using DSF using a reverse transcription-polymerase chain reaction (RT-PCR) CFX96 system (BioRad, Hercules, CA, USA). After the addition of 5× SYPRO Orange to the solutions of 0.5 μM protein without or with 200 μM inhibitor, the temperature was gradually increased from 30 to 90 °C to excite the dye at 492 nm, and the intensity of the emitted fluorescence was measured at 610 nm. The *T*_m_ value, which is the mid-point melting temperature, was calculated from a nonlinear fit of the temperature-dependent fluorescent intensity *I*(*T*), expressed as the Boltzmann equation:

$$I(T)=LL+\frac{UL-LL}{1+\exp(\frac{T_{m}-T}{a})},$$

where *UL* and *LL* are related to the baseline and top of the curves, respectively, and *a* is the steepness of the slope [63]. Only data in the range of 35–65 °C were used for the fitting.

Cell-based activity assays with inhibitors—The melanin content and MTT conversion were measured in B16F10 murine melanoma cells that were purchased from the Korean Cell Line Bank (Seoul, Korea). When the density of B16F10 cells reached 1 × 10^5^ cells, the test inhibitors (10, 20, and 50 μM) and 3-isobutyl-1-methylxanthine (IBMX, 100 μM) were added, and the cells were incubated for 48 h. The amount of melanin released into the extracellular area was quantified by measuring the absorbance at 405 nm, and was subsequently expressed as a percentage relative to the untreated control. The change in the reduction of the MTT dye into formazan in the cell was quantitatively interpreted as the cellular toxicity induced by the inhibitor. The inhibition of tyrosinase was also estimated using the B16F10 cell lysates, as described previously [28]. In brief, the cells were lysed in PBS supplemented with 1% (*w*/*v*) Triton X-100, centrifuged at 14,000 *g* for 30 min, and then the supernatants were dialysed to remove Triton X-100. After adding 500 μM L-DOPA and the inhibitors (10, 20, and 50 μM) to the prepared lysates, the change in absorbance over time at 475 nm was measured. The decrease in absorbance was calculated relative to the absorbance of the untreated controls.

## Figures and Tables

**Table 1:** Details of compounds tested quantitatively for inhibition of mushroom tyrosinase.

Chemical	2D Structure	MW	LogP ^&^	*K_i_* (μM)	LE ^#^
Thioguanine	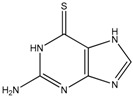	167	0.60	52	0.49
Mercaptopurine	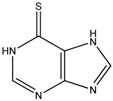	152	1.00	16	0.61
Thioinosine	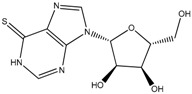	284	−0.90	8	0.34
Azathioprine	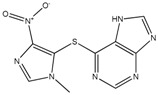	277	1.15	>1000	NA
Guanine	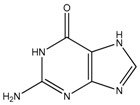	151	−0.77	>1000	NA
Hypoxanthine	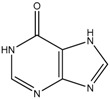	136	−0.35	>1000	NA
Kojic acid	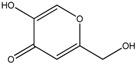	142	−0.16	13	Control

**^&^** LogP values were extracted from the ZINC database [33,34,35]. ^#^ LE, ligand efficiency is defined as −1.37 × (Log_10_*K_i_*)/*HA*, where *HA* indicates the number of heavy atoms [39].

## Supplementary Materials

Supplementary materials can be found at www.mdpi.com/1422-0067/19/1/77/s1.
