# Supplementary material for: Thiopurine Drugs Repositioned as Tyrosinase Inhibitors
Source: Int J Mol Sci. 2017 Dec 28;19(1):77. doi: 10.3390/ijms19010077 (PMC5796027; doi:10.3390/ijms19010077)
Supplement: Supplementary file 1 [file ijms-19-00077-s001.pdf]

Supplementary materials

Thiopurine Drugs Repositioned as Tyrosinase Inhibitors

Joonhyeok Choi, You-Mie Lee and Jun-Goo Jee

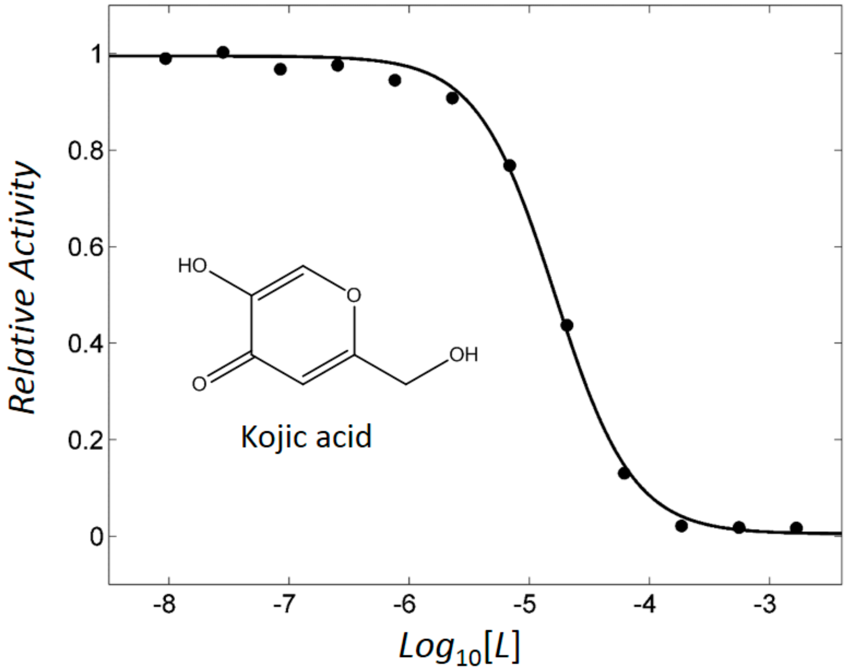

Figure S1. Profile of concentration-dependent tyrosinase inhibition by kojic acid. Activities were scaled to have a relative value in the range of 0–1.

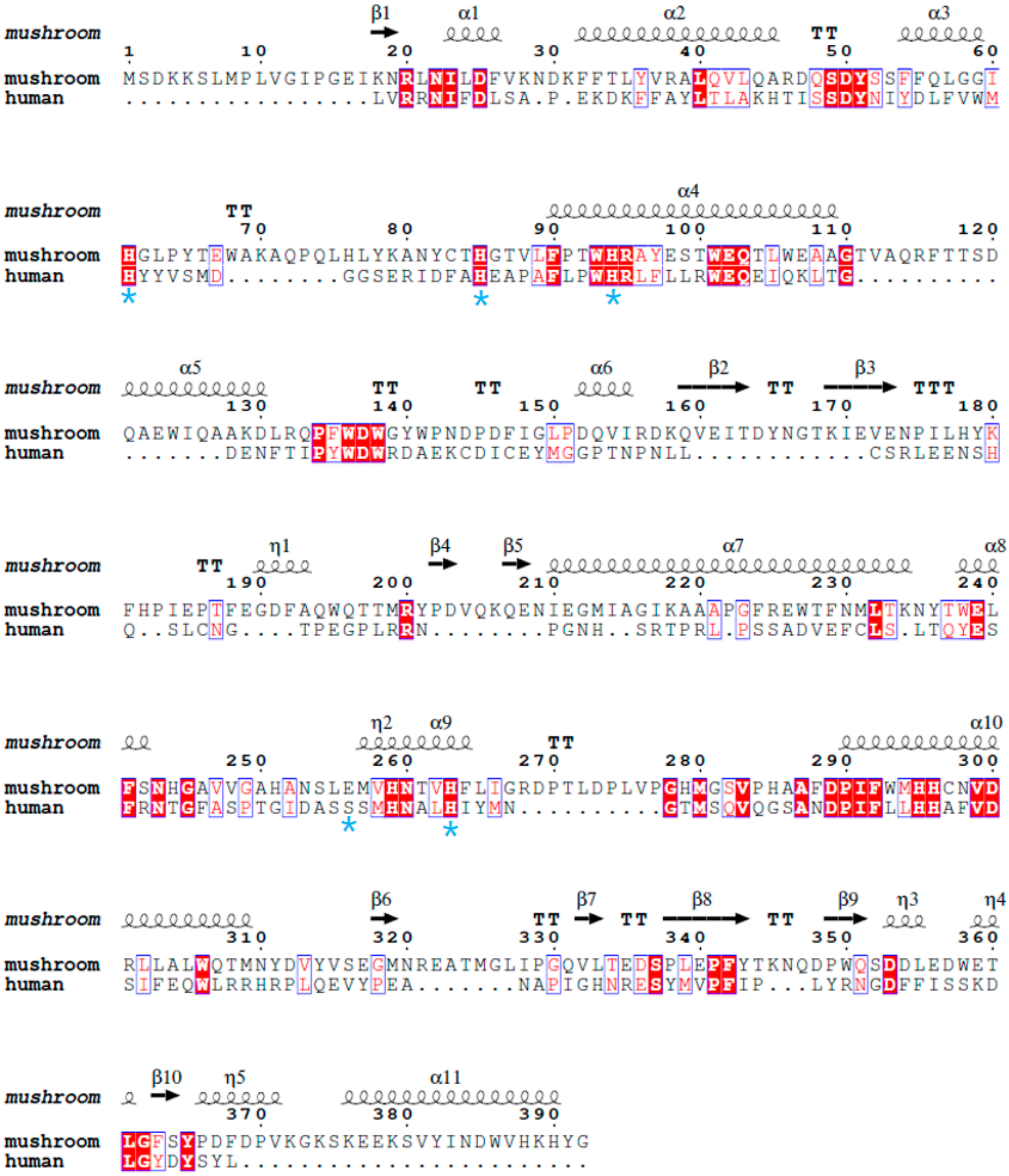

Figure S2. Sequence alignment of mushroom and human tyrosinases. I-TASSER (https://zhanglab.ccmb.med.umich.edu/I-TASSER/) generated the structure of human tyrosinase (UniProt ID: P14679). The structure-based sequence alignment between mushroom and human tyrosinases followed with TM-align (https://zhanglab.ccmb.med.umich.edu/TM-align/). Only the aligned residues in human tyrosinase are extracted for clarity. The secondary structures are drawn based on the structure of mushroom tyrosinase (PDB ID: 2Y9W). The shared contacting residues with thiopurine inhibitors in the docked models are marked with * in blue color. ESPript (http://espript.ibcp.fr/ESPript/ESPript) prepared the alignment.

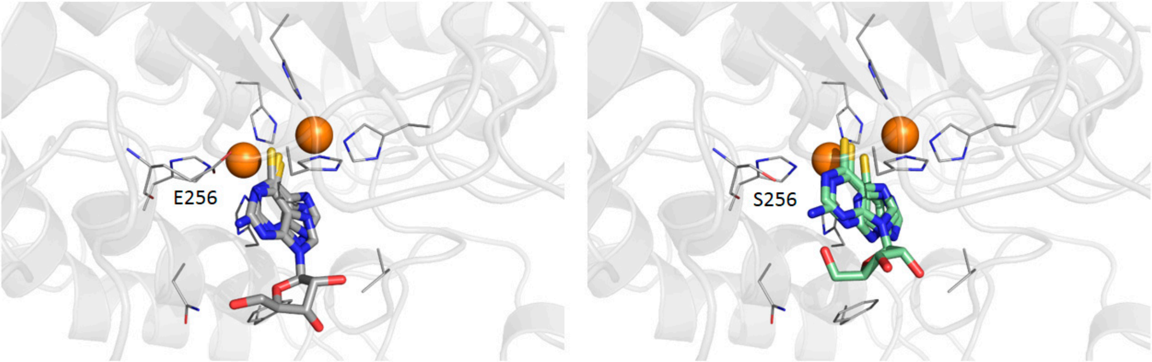

Figure S3. Docking simulation of thiopurine inhibitors with the E256S mutant. The mutant of mushroom tyrosinase, E256S, was prepared using AMBER package, not changing the geometries of the other parts except the side chain of Glu-256. The overlaid figures in the wild-type (left) and the mutant (right) have the same directions to the figures in the main text (Figure 6). Whereas the thiopurine moieties from thioguanine and thioinosine are entirely overlapped, the pose of mercaptopurine deviates in the case of the E256S mutant. Thiopurine and thioguanine in the E256S mutant contain the intermolecular hydrophilic contacts with Ser-256 within 3.8 Å whereas the contact disappears in mercaptopurine.

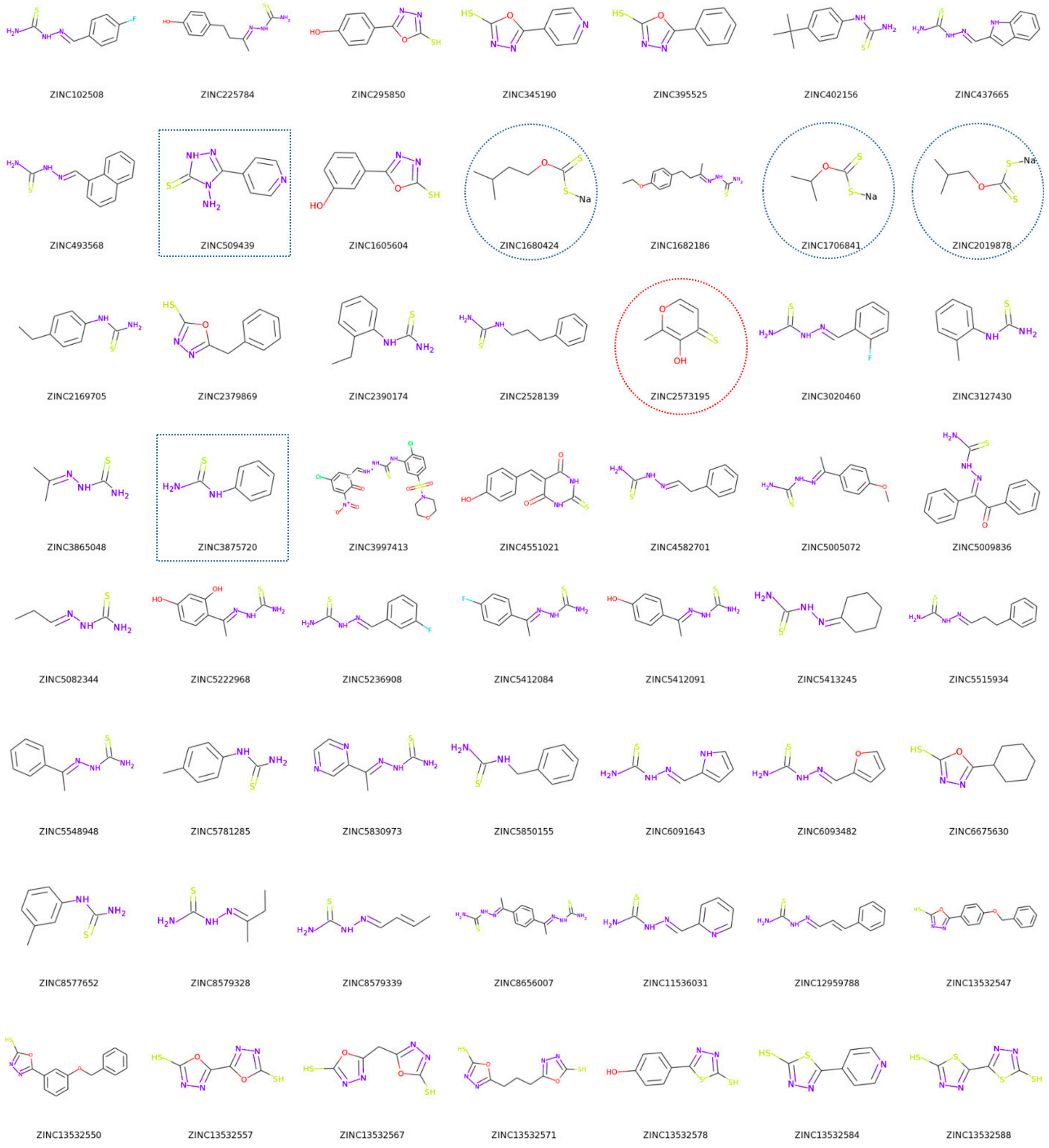

Figure S4. Thione-containing tyrosinase inhibitors. Of the 418 BindingDB-deposited known inhibitors, 115 thione-containing molecules were extracted. Four molecules possess thiones to which no nitrogen is bound are circled. One molecule (ZINC2573195) belong to a polyphenol is coloured in red. The others coloured in blue (ZINC1680424, ZINC1706841, and ZINC2019878) are xanthate molecules. Boxed inhibitors (ZINC509439 and ZINC3875720) are the molecules mentioned in the text. ZINC3875720 is phenylthiourea.

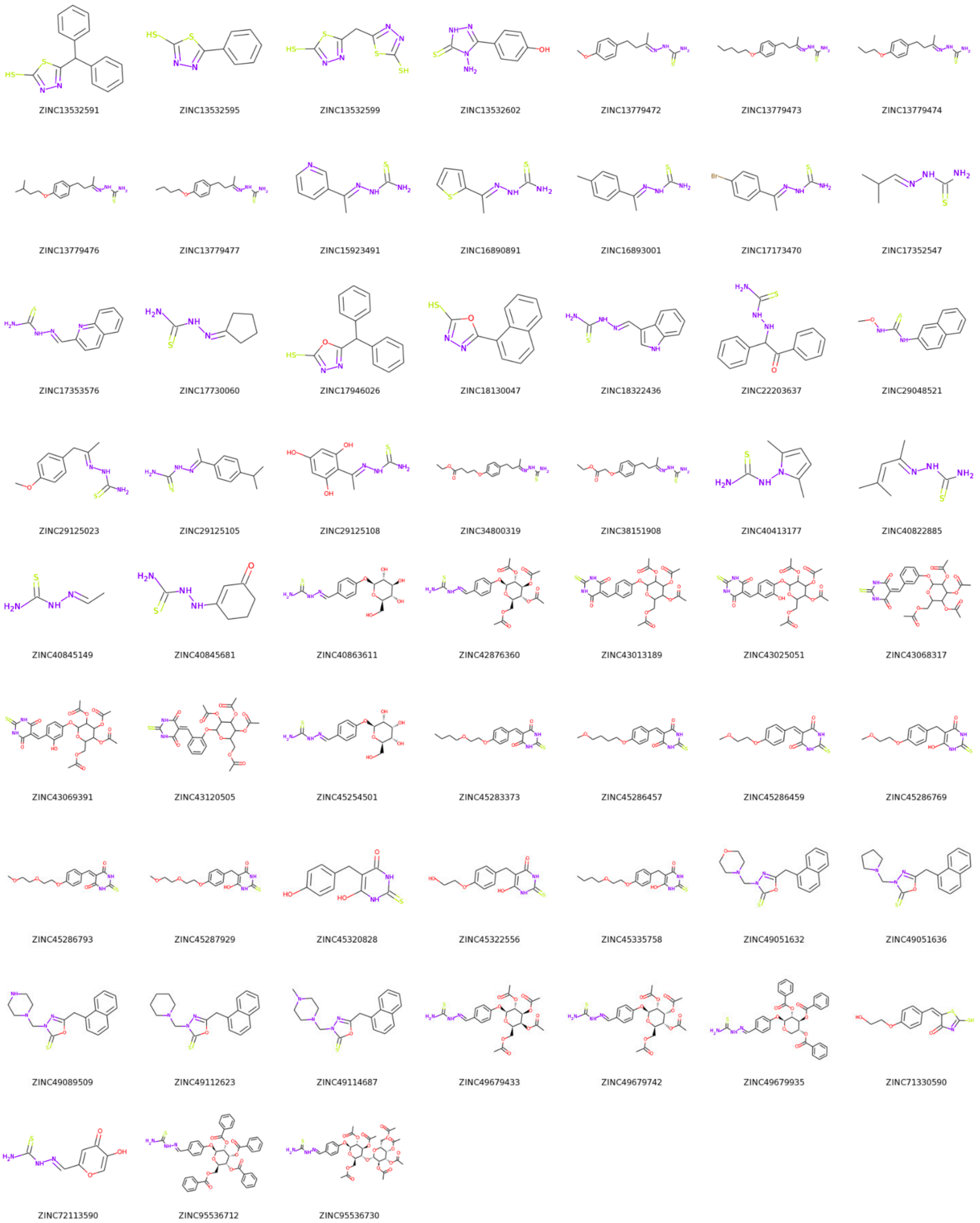

Figure S4. (continued)
